# Precise Detection of Gene Mutations in Fine-Needle Aspiration Specimens of the Papillary Thyroid Microcarcinoma Using Next-Generation Sequencing

**DOI:** 10.1155/2019/4723958

**Published:** 2019-02-19

**Authors:** Feng-xia Yu, Min-xia Hu, Han-xue Zhao, Li-juan Niu, Xue-yu Rong, Wei-hua Li, Qiang Zhu, Jian-ming Ying, Ning Lyu

**Affiliations:** ^1^Department of Diagnostic Ultrasound, Beijing Tongren Hospital, Capital Medical University, Beijing 100730, China; ^2^Department of Diagnostic Ultrasound, National Cancer Center/Cancer Hospital, Chinese Academy of Medical Sciences and Peking Union Medical College, Beijing 100021, China; ^3^Department of Pathology, National Cancer Center/Cancer Hospital, Chinese Academy of Medical Sciences and Peking Union Medical College, Beijing 100021, China

## Abstract

**Purpose:**

To assess the feasibility of next-generation sequencing (NGS) to detect mutations in BRAF, RAS, TERT promoter, and TP53 genes in ultrasound-guided fine-needle aspiration (FNA) biopsy samples of the papillary thyroid microcarcinoma (PTMC).

**Methods:**

A total of 135 FNA samples out of 135 patients with suspected PTMC were submitted for mutation testing using NGS. NGS was successfully performed in 114 specimens, while the remaining 21 samples were excluded due to insufficient amount/poor quality of DNA and sequencing failure. Of those 114 samples, 72 who were confirmed as having PTMC by postoperative histopathology were enrolled in our study, and the other 42 who had a follow-up with ultrasound were excluded. Mutations of genes including BRAF, NRAS, HRAS, KRAS, TERT promoter, and TP53 were evaluated using NGS. The associations of gene mutations and clinicopathological characteristics of PTMC were analyzed.

**Results:**

BRAF mutation was observed in 59 (81.94%) of 72 specimens. This mutation detected in BRAF was p.V600E (c.1799T>A) in exon 15 of all 59 specimens. NRAS mutation was identified in 1 (1.39%) specimen classified as Bethesda III and pathologically confirmed as a follicular variant PTMC. There were no mutations found in TERT promoter or TP53. The tumor with a maximum diameter (*D*_max_) larger than 5 mm was shown to be significantly correlated with the BRAF mutation in a multivariate analysis (OR 5.52, 95% CI 1.51-26.42, *P* = 0.033). But the BRAF mutation was not found to be significantly associated with the gender or age of patients with PTMC (*P* > 0.05).

**Conclusions:**

This study demonstrated that gene mutations in FNA specimens of PTMC could be successfully analyzed with a higher sensitivity using NGS compared to conventional methods for mutation detection. BRAF mutation of p.V600E was statistically associated with PTMC with a *D*_max_ larger than 5 mm.

## 1. Introduction

The incidence of papillary thyroid carcinoma (PTC) has been increasing in recent decades faster than any other cancer across the globe [1]. Up to 50% of these new diagnoses are the papillary thyroid microcarcinoma (PTMC), which is defined as a tumor of 1 cm or less in maximal diameter [2]. Some theses demonstrated that the PTMC had a behavior of indolent advancement. Only 1-2% of patients with PTMCs suffered from extrathyroidal invasion, regional lymph node metastasis (LNM), or recurrence from large sample size studies [3, 4]. In one cancer database, only a 0.5% disease-specific 10-year mortality was reported in 18,445 PTMC patients [5].

However, a small proportion of patients with PTMC are found to develop unfavorable outcomes. Approximately 3.5% of the tumor was reported as high-risk cancer with aggressiveness at the initial diagnosis, having cause-specific survival rates at 5, 10, and 20 years after detection being 98%, 80%, and 53%, respectively, and the recurrence rate after surgical treatment was 38% [4]. The challenge is to efficiently stratify patients according to their risk of aggressiveness to adopt surgical treatments for the lesion of high risk but active surveillance for the tumor without aggressiveness. However, it is often not an easy task to preoperatively identify tumorous aggressiveness only with sonographic appearances. In view of this issue, it is important to find some indices (biomarkers, etc.) that may precisely predict the aggressive behavior of PTMC before therapy.

Currently, genes containing mutated hotspots of thyroid cancer mainly include BRAF, RAS, TERT promoter, and TP53. The BRAF mutation, confirmed as the most common driver mutational event involved in PTC [6, 7], has been identified as a highly specific marker of thyroid cancer as the mutation occurs exclusively in thyroid malignancy rather than in benignancy [8, 9]. In addition to the BRAF mutation, point mutations of the RAS gene, including NRAS, HRAS, and KRAS isoforms [6], represent the second most common genetic alterations in PTC [10]. In recent years, studies have found that TERT promoter mutations are emerging as a feasible tool for thyroid molecular prognostication [11, 12]. Moreover, TP53 mutations may also act as a prognostic marker because it is typically considered a marker of tumor differentiation [6, 10]. In more in-depth molecular studies of PTMC, a correlation between BRAF and/or TERT promoter mutations and high-risk clinicopathological features of the lesion were described [13, 14].

The conventional methods for detecting gene mutations are direct sequencing, dual priming oligonucleotide-based multiplex polymerase chain reaction (PCR), pyrosequencing, and real-time PCR. These methods were mostly used to analyze with formalin-fixed and paraffin-embedded tissues [11, 15]. Studies on the assessment of gene mutations detected with cytological samples of PTMC, which is widely popularly used in diagnosis of thyroid nodules, have been limited in literature [16, 17]. Recently, next-generation sequencing (NGS) is becoming more widely available and has shown advantages because it is more affordable in cost and effective than other techniques with both higher sensitivity and specificity [18, 19]. Using NGS, such low mutant alleles of 5% or less can be detected only in 10 ng of genomic DNA [20]. This is particularly valuable for mutation sequencing with smaller FNA samples. Furthermore, its predominant advantage is to allow for simultaneous analysis of many mutations [18, 21]. To our knowledge, there have been no published studies to investigate the value of NGS on the assessment of mutation detection for PTMC with cytological samples.

In this prospective study, we analyzed mutations in multiple genes of BRAF, RAS, TERT promoter, and TP53 in ultrasound-guided FNA samples of patients with PTMC using NGS to provide a feasible and sensitive method of detecting gene mutations of the lesion preoperatively.

## 2. Materials and Methods

### 2.1. Study Patients

The study was approved by our institutional review board. Informed consent was obtained from all patients. Between September 2017 and July 2018, FNA procedures were performed in our department for 167 consecutive patients with suspected solitary PTMC. The risk of malignancy for thyroid nodule was classified on the basis of the Thyroid Imaging, Reporting and Data System (TI-RADS) proposed by the American College of Radiology (ACR), in which one pointing system of sonographic features was established by composition [22]. Thyroid nodule evaluated with TR4 or TR5 was considered as having a high risk of malignancy. FNA procedures were then performed according to the preference of the physician or patient [23], though active surveillance is an alternative for selective patients with suspected PTMC in our institution. The exclusion criteria of our study were as follows: the age of the patient < 18 years (*n* = 3), a history of thyroid surgery (*n* = 28), and refusal of gene sequencing (*n* = 2). Subsequently, a total of 135 FNA samples out of the 135 patients with a suspected PTMC were submitted for mutation testing using NGS. NGS was successfully performed in 114 specimens, while the remaining 21 were excluded due to insufficient amount/poor DNA quality. Finally, of those 114 samples, 72 who were confirmed as having PTMC by postoperative histopathology were enrolled in the study and the other 42 having an ultrasound (US) follow-up with Bethesda categories I-IV were excluded from the research data.

### 2.2. FNA

US examination and US-guided FNA were performed by three radiologists with more than 20 years of experience in ultrasonography of thyroid diseases. A 22-gauge fine aspiration cytology needle (Hakko Corporation Ltd., Chikuma-Shi, Nagano, Japan) was used in the FNA procedure. The needle tip with a double-cut oblique plane is identified as a bright and tiny wedge shape when the plane is oriented toward or backward to the transducer, which helps to accurately place the tip into target areas of a small lesion under US guidance. Each nodule was aspirated twice or three times within target areas being solid, hypoechoic, and relatively hypervascular on US imaging. When the needle tip was targeted into the nodule and ready for sampling, the inner needle was pulled out, and then, the needle body was drawn back and forth with a rate of 2 cycles per second. Additionally in the procedure, the needle tip was slightly retracted, merely adjusted in different puncture directions, and inserted into a different target area so that a multidirectional sampling might be performed in the nodule measured larger than 5 mm. Aspirates were sprayed onto glass slides and immediately fixed in 95% alcohol. The alcohol-fixed smears were subsequently stained with haematoxylin and eosin (H&E) for cytological diagnosis. Cytological results were obtained according to The Bethesda System for Reporting Thyroid Cytology (TBSRTC) [24]. After acquisition of the optimal quantity for cytological diagnosis, residual biopsy aspiration was sprayed into a sterile 1.5 mL Eppendorf tube, stored immediately in dry ice below minus 60°C, and then sent to the laboratory within 2 hours for immediate DNA extraction.

### 2.3. Isolation of Genomic DNA

DNA was extracted from cytology specimens using Autostation Cells/Tissue Genomic DNA Kits (ACCB Biotech Ltd., Beijing, China). The NanoDrop ND-1000 Spectrophotometer (NanoDrop, Waltham, MA, USA) was used to evaluate DNA quality. The Qubit™ Fluorometer (Thermo Fisher Scientific Inc., Waltham, MA, USA) was used to determine DNA quantity.

### 2.4. DNA Library Construction and Sequencing

Amplicon libraries were prepared using 10 ng of genomic DNA with the Ion AmpliSeq Thyroid Panel (Thermo Fisher). There were 6 cancer-related genes included in the custom panel: BRAF, NRAS, HRAS, KRAS, TERT promoter, and TP53. The amplicon libraries were constructed with an Ion AmpliSeq Library Kit 2.0 (Thermo Fisher), following the manufacturer's protocols. Next, the libraries were quantified using the Ion Library Quantification Kit (Thermo Fisher). Then, each library was diluted to a concentration of 100 pM and pooled in equal volumes. Emulsion PCR and template preparation were performed to get the template-positive ion sphere particles (ISPs). Finally, ISPs were sequenced on an Ion S5 (Thermo Fisher) using the Ion 520 Kit-Chip (Thermo Fisher).

### 2.5. Data Analysis and Validation

When the total reads were >300,000 and AQ20 > 98% (1 misaligned base per 100 bases), the sequencing was identified successfully. Mutations were annotated through the Torrent Variant Caller (Thermo Fisher) and viewed using the Integrative Genomics Viewer (Thermo Fisher). Variants with ≥5% mutant allele frequency and >1000× coverage were considered true variations.

### 2.6. Statistical Analysis

All data were analyzed using SPSS 20.0. Continuous variables were expressed as the mean ± standard deviation (SD), and categorical variables as the number of cases and percentage (%). Differences between groups were tested with Student's *t*-test and the chi-square test or Fisher's exact test according to continuous variables or categorical variables, respectively. Multivariate logistic regression analysis was used to determine the clinicopathological factors independently associated with the BRAF mutation. A 2-sided *P* value < 0.05 was considered to indicate a statistically significant difference.

## 3. Results

### 3.1. General Characteristics

The ages of the 72 patients (19 men and 53 women) were 42.46 ± 11.92 years (range, 25-71 years). According to the recommendation of the American Joint Committee on Cancer (AJCC) 8th edition [25], the patients were divided into subgroups of ages less than 55 years (*n* = 60) and 55 years or older (*n* = 12). The sizes (maximum diameter, *D*_max_) were 7.19 ± 2.01 mm (range, 3.2-10.0 mm) measured on the sonogram. The tumors were then classified as tumors with a *D*_max_ of 5 mm or less (*n* = 8) and tumors with a *D*_max_ larger than 5 mm (*n* = 64). Detailed composition of these 72 specimens is shown in Table 1.

### 3.2. Mutation Profiling of the PTMC

**Figure 1:**
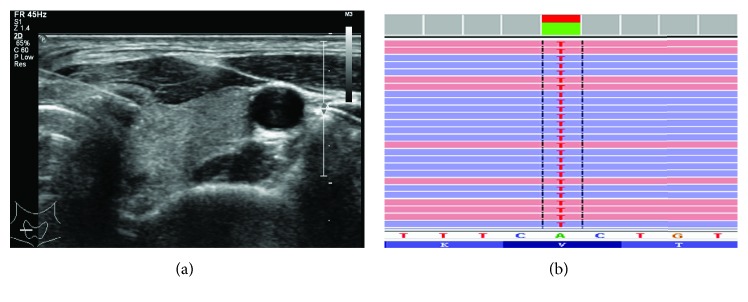
Sonogram of US-guided FNA biopsy for a 36-year-old woman with PTMC (a). NGS of the FNA sample showed a BRAF p.V600E (c.T1799A) mutation (b).

**Figure 2:**
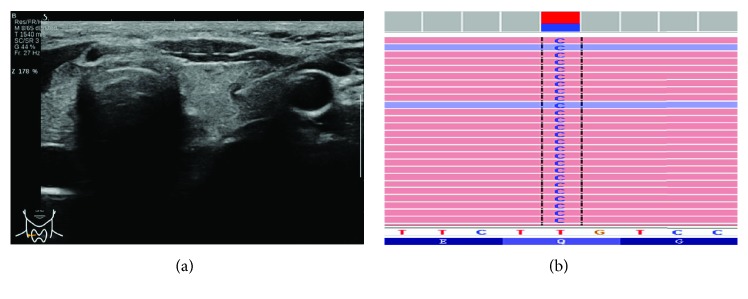
Sonogram of US-guided FNA biopsy for a 33-year-old woman classified as Bethesda III in cytological diagnosis and subsequently pathologically confirmed to have a follicular variant of PTMC (a). NGS of the FNA sample showed an NRAS p.Q61R (c.A182G) mutation (b).

BRAF mutation was observed in 59 (81.94%) of 72 specimens. The mutation detected in BRAF was p.V600E (c.1799T>A) in exon 15 of all 59 specimens (Figure 1). NRAS mutation was identified in only 1 (1.39%) specimen classified as Bethesda III, which is p.Q61R (c.182A>G) in exon 3 (Figure 2). This patient was pathologically confirmed having a follicular variant of PTMC. There were no mutations found in TERT promoter or TP53. The relationship between the BRAF^V600E^ mutation and TBSRTC is listed in Table 1.

### 3.3. Relationship between the Factors of Gender, Age, and *D*_max_ and BRAF Mutation

In the positive and negative groups of BRAF mutation, the ages were 41.53 ± 12.36 years (range, 25-71 years) and 46.69 ± 8.87 years (range, 27-60 years) and the *D*_max_ of the tumors were 7.19 ± 1.93 mm (range, 3.2-10.0 mm) and 7.23 ± 2.46 mm (range, 4.2-10.0 mm), respectively. The differences in gender, age, and *D*_max_ of the tumors between the two groups are listed in Table 2. *D*_max_ was significantly associated with the BRAF mutation in univariate analyses (*P* = 0.045). Multivariate analyses further verified that PTMC with a *D*_max_ larger than 5 mm was significantly correlated with mutated BRAF (OR 5.52, 95% CI 1.51-26.42) (Table 3). The BRAF mutation was not statistically associated with the gender or age of patients with PTMC (*P* > 0.05).

### 3.4. The *D*_max_ of the Nodules Associated with Failed Sequencing

There was a significant difference between the *D*_max_ values of 21 nodules with failed sequencing (4.95 ± 1.20 mm; range, 3.4-8.2 mm) and those of the 114 nodules with successful sequencing (7.48 ± 1.97 mm; range, 3.2-10.0 mm) (*P* = 0.001). In addition, there was a higher proportion of nodules with a *D*_max_ of 5 mm or less in the sequencing failure group compared with that of the successful sequencing group (42.86% vs. 10.53%, *P* = 0.001).

## 4. Discussion

Compared to conventional methods of testing for mutations, NGS has been proven to be able to detect multiple gene mutations with higher sensitivity and specificity using less input DNA at a relatively lower cost [26]. To our knowledge, the present study is the first report in literature that attempts to explore the application of NGS in the detection of multiple gene mutations of PTMC using FNA samples. In this study, we investigated 72 US-guided FNA samples of PTMC using NGS to detect mutations in 6 cancer-related genes. It was found that gene mutations in FNA specimens of PTMC could be successfully analyzed using NGS with a higher sensitivity than that of other techniques, particularly in larger lesions.

BRAF mutation, which has been relatively thorough studied, can activate the MAPK pathway resulting in intense cellular proliferation, inhibition of differentiation, and apoptosis. The most common alteration is a point mutation at codon 600 [27]. According to the Thyroid Cancer Genome Atlas (TCGA), BRAF^V600E^ largely represents the most common driver mutational event involved in PTC [6]. A study that analyzed 339 PTMC cytological samples in Korean patients found that 213 (62.8%) had BRAF mutation detected with direct sequencing [13]. A study that included FNA samples of Chinese patients with PTMC using PCR showed that the frequency of the BRAF mutation in the PTMC was 34% (21/61) [28]. A recent study of solitary PTMC in 214 Chinese patients revealed that the incidence of the BRAF mutation was 82.2% (176/214) by real-time fluorescent PCR detection with paraffin-embedded postoperative tissue samples [29]. In this study, it was found that using NGS, the mutated BRAF was present in up to 81.94% of FNA samples of PTMC. This result indicated that the prevalence of mutated BRAF detected for cytological samples of PTMC using NGS was nearly consistent with that of conventional sequencing methods for postoperative histological tissues, and that NGS had a higher sensitivity than conventional sequencing for analysis of FNA samples. Our study suggested that NGS might be suitable for cytological samples of the PTMC and achieve higher sensitivity using less DNA.

As the second most common genetic alteration in PTC, point mutations of the RAS gene result in the constitutive and aberrant activation of downstream MAPK and PI3K/AKT signaling pathways, which is a critical event in thyroid tumorigenesis [30, 31]. RAS alterations can occur in 12.7% (8.5% NRAS, 3.5% HRAS, and 0.7% KRAS) of the PTC cohort, 40-50% of follicular thyroid carcinoma (FTC), and 20-25% of follicular adenomas [10]. Adeniran et al. found that almost all of the PTCs harboring RAS mutations were classified as the follicular variant [32]. Similarly, our study found that 1 (1.39%) of 72 specimens classified as Bethesda III in the cytological diagnosis had mutated NRAS. This patient was consequently confirmed as having a follicular variant of PTMC. Recently, as emerging molecular prognosticators, TERT promoter mutations have attracted a considerable amount of interest. Mutations of the TERT promoter induce the formation of a consensus binding site for ETS (E-twenty-six) transcription factors that leads to increased gene expression, thus inducing telomerase activation, inhibition of the physiological telomere shortening, and immortalization of cancer cells [33, 34]. It has been recognized that TERT promoter was more frequently detected in poorly differentiated and undifferentiated thyroid cancers [10]. There were no mutations found in TERT promoter in the study. But the result was different to of de Biase and coworkers, in which gene mutations were detected in 4.7% (19/404) of patients with PTMC in Italy [35]. Coincidentally and interestingly, it was reported that only 2.0% (7/355) of PTC cases were found to harbor TERT promoter mutations in a large-scale genetic analysis in Chinese patients to determine a mutational landscape, and the frequency was significantly lower than one of Americans (2.0% vs. 9.4%, *P* < 0.001) [6, 31]. Accordingly, it was suggested that the prevalence of TERT mutations in PTMC varies probably in diverse races and environment factors, etc. Moreover, no detection of TERT promoter mutations in our results could be related to the smaller sample size. TP53 gene mutations considered as markers of tumor dedifferentiation play a critical role in DNA damage response, differentiation, proliferation, and death of the cell. It is a tumor suppressor that is inactivated in half of all human cancers, and thus, mutated TP53 can induce tumorigenesis [36]. All the nodules analyzed were well-differentiated PTMCs, which could explain that there was no TP53 mutation detected in the present study.

The association between the factor, including gender, age, or tumor size, and the BRAF mutation in PTMC has been unclear [37]. In addition, as far as we know, there have been few documents related to the analysis of these three factors in PTMC. We found that the BRAF mutation was not associated with neither the gender nor age of patients (*P* > 0.05), which was consistent with the result published by Kwak et al. [13] and Sun et al. [17]. However, the study of Zheng et al. [16] showed a significant relationship between the BRAF mutation and male gender (OR 1.83, 95% CI 1.02-3.69), but not age, using multiple logistic regression analysis. It was considered that the proportion of male patients and the total sample size might affect the results in various studies. In consistent with the viewpoint of Kwak et al. [13], our results showed that the *D*_max_ of the nodules was associated with the BRAF mutation while Zheng et al. [16] and Sun et al. [17] suggested that mutated BRAF was not related to the size of PTMC. The possible reasons for the differences were sample size, distribution of nodular size, specimens (paraffin-embedded tissues or FNA samples), techniques of detecting gene mutations, and the difference of the clonal/subclonal status of mutated BRAF [38]. In further analysis of our data, it was found that only 4 (50.0%) of 8 nodules with a *D*_max_ of 5 mm or less had mutated BRAF, but this mutation occurred in 55 (85.9%) of 64 nodules with a *D*_max_ larger than 5 mm. Compared with the successfully sequenced nodules, the *D*_max_ of the nodules that failed sequencing was smaller (*P* = 0.001), and a higher proportion of nodules with a *D*_max_ of 5 mm or less was found in the sequencing failure group (42.86% vs. 10.53%, *P* = 0.001). These results implied that gene sequencing, including NGS, could have certain limitations in cytological samples for thyroid nodules with a *D*_max_ of 5 mm or less. Further comparable studies are needed to support and interpret this relationship further in depth and to explain the differences in the results.

There are some limitations in our study. Firstly, the nodule with a unitary subtype of the tumor was studied, including differentiated papillary carcinomas rather than FTC and poorly differentiated and undifferentiated thyroid cancers. Secondly, the sample number was limited, which would have impacted the detection of mutations other than BRAF mutation. And finally in the future, a study with a large sample size of PTMC patients is needed to observe the relationship between gene mutations and prognostic factors including LNM, extrathyroidal extension, and tumor recurrence.

## 5. Conclusion

In conclusion, this study demonstrated that gene mutations in FNA specimens of the PTMC could be successfully analyzed using NGS with higher sensitivity than that of conventional methods of mutation detection. The BRAF mutation was statistically associated with PTMC with a *D*_max_ larger than 5 mm, but neither the gender nor the age of the PTMC patients. However, due to the small diameter of the nodule, the value of NGS for cytological samples of nodules with a *D*_max_ of 5 mm or less was questionable.

## Figures and Tables

**Table 1:** The associations of the BRAF mutation and the Bethesda categories in 72 specimens.

BRAF mutation	Bethesda categories					
	I	II	III	IV	V	VI
Positive	2	0	3	0	23	31
Negative	0	0	1	0	7	5
Total	2	0	4	0	30	36

**Table 2:** The relationship between clinicopathological parameters and BRAF mutation in PTMC.

Factors	BRAF mutation		*P* value
	Positive (*n*, 59, 81.9%)	Negative (*n*, 13, 18.1%)	
Gender			0.518
Male	17	2	
Female	42	11	
Age (years)			0.784
<55	50	10	
≥55	9	3	
Maximum diameter (mm)			0.045
≤5.0	4	4	
>5.0	55	9	

**Table 3:** Multivariate logistic regression analysis of factors independently associated with BRAF mutation in PTMC.

Factors	OR (95% CI)	*P* value
Gender	0.508 (0.095-2.717)	0.429
Age (years)	0.646 (0.137-3.041)	0.581
Maximum diameter (mm)	5.516 (1.151-26.422)	0.033

## Data Availability

The data used to support the findings of this study are available from the corresponding author upon request.
